# Sex Differences between CRF1 Receptor Deficient Mice following Naloxone-Precipitated Morphine Withdrawal in a Conditioned Place Aversion Paradigm: Implication of HPA Axis

**DOI:** 10.1371/journal.pone.0121125

**Published:** 2015-04-01

**Authors:** Juan-Antonio García-Carmona, Alberto Baroja-Mazo, María-Victoria Milanés, María Luisa Laorden

**Affiliations:** 1 Department of Pharmacology, School of Medicine, University of Murcia, Murcia, Spain; 2 Group of Inflammation, FFIS-University Hospital V. A., Murcia, Spain; Radboud University, NETHERLANDS

## Abstract

**Background:**

Extinction period of positive affective memory of drug taking and negative affective memory of drug withdrawal, as well as the different response of men and women might be important for the clinical treatment of drug addiction. We investigate the role of corticotropin releasing factor receptor type one (CRF1R) and the different response of male and female mice in the expression and extinction of the aversive memory.

**Methodology/Principal Finding:**

We used genetically engineered male and female mice lacking functional CRF1R. The animals were rendered dependent on morphine by intraperitoneally injection of increasing doses of morphine (10–60 mg/kg). Negative state associated with naloxone (1 mg/kg s.c.)-precipitated morphine withdrawal was examined by using conditioned place aversion (CPA) paradigm. No sex differences for CPA expression were found in wild-type (n = 29) or CRF1R knockout (KO) mice (n = 29). However, CRF1R KO mice presented less aversion score than wild-type mice, suggesting that CRF1R KO mice were less responsive than wild-type to continuous associations between drug administration and environmental stimuli. In addition, CPA extinction was delayed in wild-type and CRF1R KO male mice compared with females of both genotypes. The genetic disruption of the CRF1R pathway decreased the period of extinction in males and females suggesting that CRF/CRF1R is implicated in the duration of aversive memory. Our results also showed that the increase in adrenocorticotropic hormone (ACTH) levels observed in wild-type (n = 11) mice after CPA expression, were attenuated in CRF1R KO mice (n = 10). In addition, ACTH returned to the baseline levels in males and females once CPA extinction was finished.

**Conclusion/Significance:**

These results suggest that, at least, CPA expression is partially due to an increase in plasma ACTH levels, through activation of CRF1R, which can return when CPA extinction is finished.

## Introduction

A growing body of evidence indicates that there are differences between men and women in the vulnerability to drug abuse [1]. While there are abundant findings indicating that females are more motivated than males during several phases of drug addiction [2–5]; there are limited data regarding sex differences in the emotional signs of withdrawal from drugs of abuse. Some studies have found increased physical signs of withdrawal in males from drugs such as ethanol [6], morphine [7], pentobarbital [8], and methaqualone [9]. Similar findings have been also reported for phencyclidine withdrawal in male versus female monkeys [10] and for alcohol withdrawal in men versus women [11]. Additionally, somatic opioid withdrawal is greater in male than in female rats and mice [7,12].

The induction of the acoustic startle reflex and conditioned place aversion (CPA) has been used to assess the emotional component of opioid withdrawal [13]. These animal models mimic the behaviors of the negative affective component of the withdrawal syndrome. CPA has been widely used to assess dysphoric or aversive aspects of withdrawal [15] and to demonstrate that the expression of opioid withdrawal relies on structures of the extended amygdale and mesolimbic dopamine system [13,14]. The physical component of the withdrawal syndrome was also assessed by scoring somatic withdrawal signs after morphine exposure [13]. Although there is increasing evidence suggesting sex differences in behavioral response to drug of abuse, the mechanism underlying these differences is currently not well understood, however it is important to guide the treatment strategy for men and women.

All major drugs of abuse stimulate the hypothalamic-pituitary-adrenocortical (HPA) axis, during acute withdrawal via the activation of corticotropin-releasing factor (CRF) in the paraventricular nucleus (PVN) of the hypothalamus, with a common response of elevated adrenocorticotropic hormone (ACTH) and corticosterone [16], which mediate somatic and negative affective-like components of withdrawal [17–20]. CRF exerts its actions through activation of two different types of G-protein-coupled receptors: CRF1 and CRF2, which are distributed throughout the periphery and the brain [21,22]. CRF1 receptor (CRF1R) has been located in several key brain areas involved in reward, reinforcement, craving and aversive effects of drugs of abuse [23]. Moreover, the decreased function of the brain reward system during drug withdrawal is CRF1R-dependent [24,25]. Despite the extensive research supporting the role of CRF in drug addiction, a scant work has been carried out to evaluate the implication of CRF receptors exploring sex differences in behaviours related to substance of abuse. Currently, no studies have been published comparing sex differences in morphine-withdrawn CRF1R deficient mice after a CPA paradigm. Here we have evaluated the role of CRF1R in 1) in the expression and extinction of CPA; 2) in the physical signs of withdrawal; and 3) in the HPA activation following CPA expression and extinction using genetically engineered male and female mice lacking functional CRF1R.

## Material and Methods

### Ethics Statement

All animals received humane care according with the guidelines provided by the European Communities Council Directive of 22 September 2010 (2010/63/UE) and were approved by the Comité Ético de Experimentación Animal (CEEA, Universidad de Murcia, RD 53/2013).

### Animals

Adult male and female B6,129 CRHtklee mice (8–12 weeks old, 25–30 g) that were wild-type (CRF1R+/+) and recessive homozygous (CRF1R−/−) or knockout (CRF1R KO) were used in these experiments. Mice were housed 4–6/standard cage in a temperature-controlled environment, received *ad libitum* access to food and water and were maintained on a 12-h/12-h light/dark cycle. Mice were habituated to the testing room for at least 1 week prior to the experimental manipulations.

CRF1R+/+ and CRF1R mutant mice were obtained from heterozygote breeding pairs, which were originated from the individual crossing of a number of heterozygote couples purchased from an external supplier (Jackson Laboratories, California, USA). All mice were genotyped at weaning using a polymerase chain reaction (PCR) based method. Tail tip samples were collected at 3 weeks of age, and DNA was extracted and processed according to the manufacturer's instructions (Qiagen, Germany).

### Drug treatment

For each sex, wild-type (WT) and CRF1RKO mice were randomly divided into two groups: chronic saline-treated (n = 42) and chronic morphine-treated (n = 47). Morphine was injected intraperitoneally (i.p.) in the mice’s home-cages with a chronic escalating-dose regimen, every 12 h (at 8 am and 8 pm), starting on day 1, 10 mg/kg; day 2, 30 mg/kg; day 3, 50 mg/kg and day 4, 60 mg/kg (only one injection in the morning). This pattern of morphine administration, which involves ascending drug doses, has been used extensively to study opioid tolerance and dependence [26–29]. The doses of morphine were selected based in previous studies from our laboratory performed in CRF1R knockout mice [30,31]. The chronic saline-treated groups were administered with saline (i.p.) using the same protocol.

### Naloxone-induced conditioned place aversion

**Fig 1:**
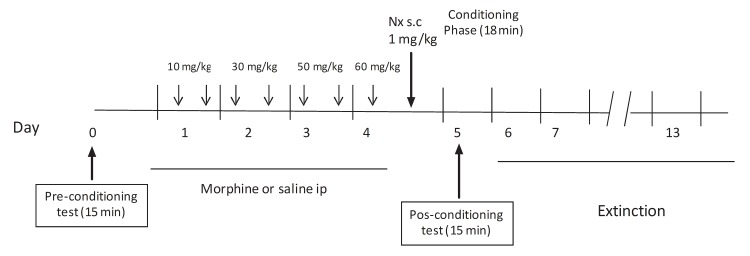
Experimental schedule for the conditioned place aversion training. Mice were treated with a chronic escalating-dose morphine regimen for 4 days in the conditioning period. On day 4, 1 h later last morphine injection, naloxone (1mg/kg, s.c.) was administered. We examined the extinction of naloxone CPA testing for aversion from day 6 to 13.

CPA is a recognized paradigm of negative affective learning. The present CPA procedure induces place aversion when the animals relate the environment with the negative effects of morphine withdrawal syndrome [31]. The CPA equipment used in this study was two identical boxes with three polyvinylcarbonate (PVC) chambers [32] connected to a computer. Two large side chambers (20 length, 18 cm width and 25 cm height) were separated by a smaller chamber (20 cm, length 7 cm width and 25 cm height). The two larger chambers differed in their wall paint and floor texture (i.e., grey striped wall with black smooth floor or black spotted wall with grey rough floor, respectively) and provided distinct contexts that were paired to naloxone injections. Three distinct chambers were separated by manual guillotine doors which were removed during the test. The CPA training procedure was used in previous experiments [31,33]. The protocol consisted of five phases: pre-test, drug treatment, conditioning, pos-test and extinction (Fig 1).

In the pre-test (day 0), the mice were placed in the middle chamber and allowed to shuttle between the three chambers in the apparatus for 15 min. The time spent in each chamber was recorded, and the animals that spent less that 390 s in either chamber were considered not to be neutral in preference for either side and were excluded from further study (n = 4). During days 1–4 animals were treated with morphine or saline as described above. On day 4 and 1 h later last morphine injection, naloxone was administered subcutaneously to all the groups at a dose of 1 mg/kg, in order to precipitate the morphine withdrawal syndrome and the mice were immediately confined to one of the chambers during 18 min and recorded to analyze naloxone-induced morphine withdrawal signs. On Day 5, the CPA expression was tested in a drug-free state (pos-test). The testing procedure was the same as the pre-test. The CPA score represents the time in the drug naloxone-paired chamber during the testing phase minus that during the preconditioning phase.

### Behaviour effects by naloxone precipitated morphine withdrawal

Videos were assessed later by an observer blind to the experimental conditions for freezing behavior, a typical defense response which is characterized by the cessation of all movement except that required for breathing, diarrhoea events, number of grooming, rubbings, rearing and escape jumps, a well characterized behavioral outcome of naloxone-precipitated morphine withdrawal [34].

### Extinction of conditioned place aversion

Briefly, extinction training began 24 h after the post-training test. The CPA extinction was performed for 7 consecutive days (day 6–13) under identical conditions of the pre-test and pos-test. Animals that displayed less than 120 s of aversion were considered to be neutral in preference and therefore to extinct the aversion.

### Radioimmunoasay

Sixty minutes later the end of the pos-test or the CPA extinction, mice were decapitated by guillotine at the same time (10:00–11:00 h) and blood samples were collected. Plasma ACTH and corticosterone concentrations were measured by using commercially available kits for mice (125 I-ACTH and 125 I-corticosterone radioimmunoassay; MP Biomedicals, USA). The sensitivity of the assay was 5.7 pg/ml for ACTH and 7.7 ng/ml for corticosterone.

### Drugs and reagents

Morphine HCl (Alcaliber, Madrid, Spain) and naloxone HCl (Sigma Chemical, St. Louis, MO, USA) were dissolved in physiological saline and all injections were administered in a volume of 0.01 ml/g body weight.

### Statistical analysis

Three-way ANOVA for the factors treatment, genotype and sex was used to compare all the data showed in this work. Moreover, repeated-measures ANOVA was performed for the analysis of the extinction of aversion and *post hoc* Bonferroni analysis was used to determine differences between treatment groups. Statistical significance was set at α level of *p*< 0.05. All data were analyzed using the SPSS statistics 19 software (IBM, USA), and are presented as mean ± SEM.

## Results

### Body weight gain after morphine administration

**Fig 2:**
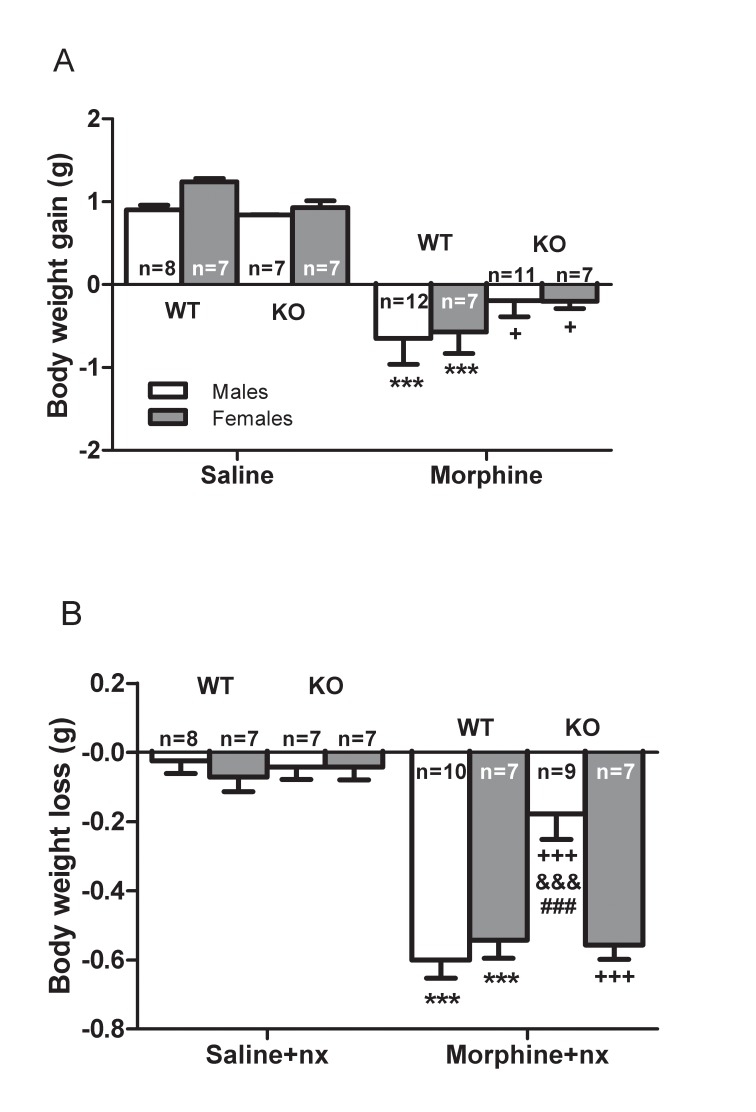
Changes in body weight. (A) Effect of saline or morphine injection on body weight in wild-type (WT) or knockout (CRF1R KO) mice. The animals received increasing doses of morphine (10–60 mg/kg, i.p.) or saline every 12 hours during four days. (B) Effect of naloxone (nx, 1mg/kg, s.c.) injection on body weight loss in WT and KO mice treated with morphine or saline. Data are the mean ± SEM. ***p<0.001 versus WT mice treated with saline, or saline+nx; +*p<*0.05 versus KO mice treated with saline or saline+nx; &&&*p<*0.001 versus male of WT mice treated with morphine+nx; ###*p<*0.001 versus female of KO mice treated with morphine+nx.

The weight of the animals was recorded the days of morphine or saline injection since it is known that chronic morphine treatment induces a decrease in body weight gain due to a lower caloric intake [35]. Three-way ANOVA for body weight gain revealed a main effect of chronic morphine treatment (F_(1,28)_ = 83.146, *p<*0.0001) but there is not significant effects of genotype (F_(1,28)_ = 0.574, *p* = 0.452) and sex (F_(1,28)_ = 0.675, *p* = 0.415). There is not significant interaction between genotype x morphine treatment (F_(1,28)_ = 3.933, *p* = 0.52), sex x genotype (F_(1,28)_ = 0.324, *p* = 0.572), sex x morphine treatment (F_(1,28)_ = 0.351, *p* = 0.556) and sex x genotype x morphine treatment (F_(1,28)_ = 0.78, *p* = 0.781). Fig 2A shows body weight gain after morphine treatment. Wild-type and CRF1R KO mice receiving morphine treatment had a significantly (*p<*0.001, p<0.05, respectively) lower body weight gain than animals receiving saline injection. Present results are in agreement with previous studies [19] which demonstrated that genetic disruption of the CRF/CRF1R circuitry did not affect the reduction of body weight gain induced by escalating morphine doses.

### Body weight loss after naloxone-precipitated morphine withdrawal

Since body weight loss induced by naloxone-precipitated morphine withdrawal is unbiased and accurate measurable sing of opioid withdrawal [3,36] we have evaluated the implication of CRF1R in this parameter. Loss of body weight was calculated as the difference between the body weight determined immediately before saline or naloxone injection and a second determination 20 min later, immediately after the conditioning. Three-way ANOVA revealed significant main effects on body weight loss for chronic morphine treatment (F_(1,28)_ = 132.754, *p<*0.0001), sex (F_(1,28)_ = 6.275, *p* = 0.015) and genotype (F_(1,28)_ = 8.093, *p* = 0.006). In addition, there was an interaction between the factors of genotype x sex (F_(1,28)_ = 7.026, *p* = 0.011), genotype x morphine treatment (F_(1,28)_ = 7.285, *p* = 0.009), and sex x genotype x morphine treatment (F_(1,28)_ = 10.769, *p* = 0.002). Naloxone injection to wild-type and CRF1RKO morphine-treated mice produced a significant (*p<*0.001) increase in body weight loss in male and female animals. However, the weight loss in morphine withdrawn-CRF1RKO male was significantly (*p<*0.001) lower than that observed in wild-type male and female KO mice after naloxone-precipitated morphine withdrawal. In addition, there was not significant differences in female wild-type and KO mice (Fig 2B).

These results indicate that female CRF1R KO mice have more severe withdrawal than males CRF1R KO mice.

### CPA to morphine withdrawal

**Fig 3:**
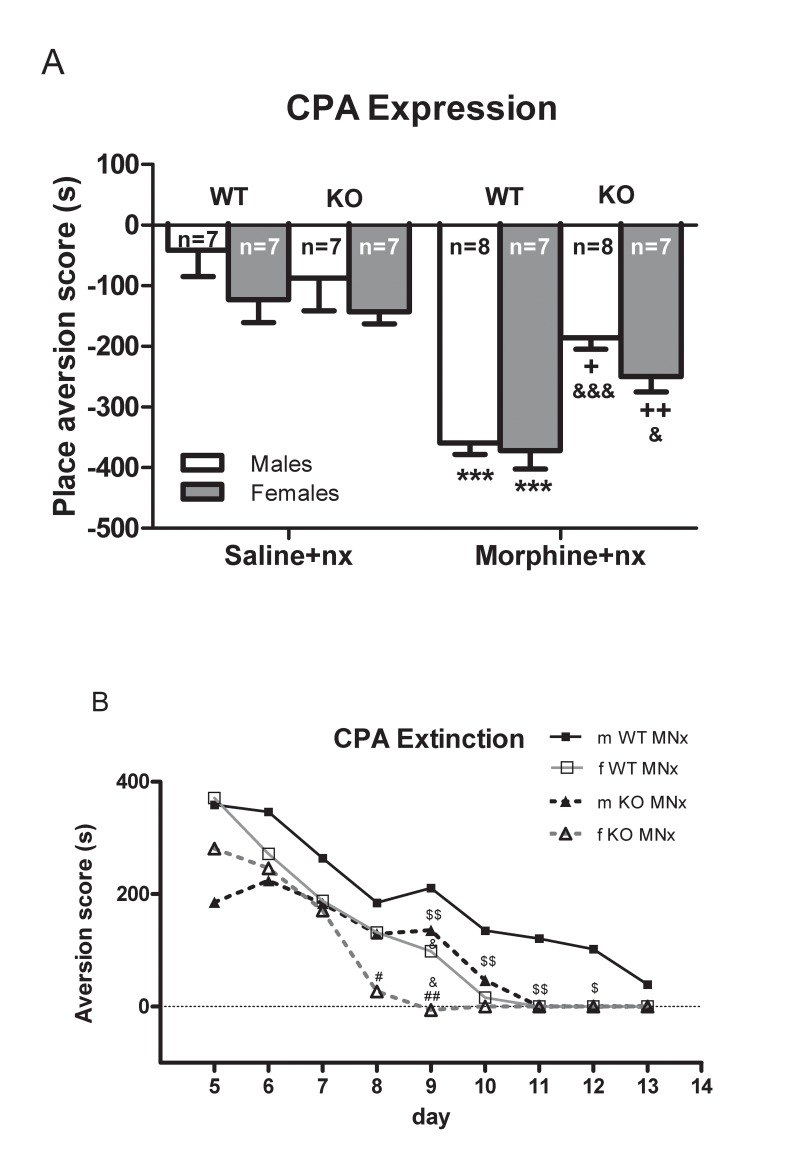
Conditioned place aversion (CPA) of morphine withdrawal. A) Expression of CPA induced by naloxone (nx, 1 mg/kg s.c.) in wild-type (WT) or knockout (CRF1R KO) mice treated with morphine or saline. The score was calculated for each mouse as the difference between the postconditioning and the preconditioning time spent in the drug-paired compartment. B) Extinction of CPA training. Aversion scores from day 5 to 13 for male (m) and female (f) from WT and CRF1R KO are shown. Data are expressed as the mean ± SEM. ***p<0.001 versus WT mice treated with saline+naloxone; +*p<*0.05, *++p<*0.01&*p<*0.05 versus KO mice treated with saline+nx, &*p<*0.05, &&&*p<*0.001 versus WT mice; #*p<*0.05, ##*p<*0.01 versus male of CRF1R KO mice; $$*p<*0.01 versus male of WT mice.

Three-way ANOVA examined the effects of genotype, sex and morphine on place aversion induced by naloxone-precipitated morphine withdrawal. We observed significant main effects of genotype (score: F_(1,28)_ = 4.696, *p* = 0.035), morphine treatment (score: F_(1,28)_ = 77.522, *p<*0.0001) and sex (score: F_(1,28)_ = 7.206, *p* = 0.010), and significant interaction between genotype x morphine treatment (score: F_(1,28)_ = 13.114, *p* = 0.001). The Bonferroni *post hoc* test showed that naloxone induced a significant (p<0.05, p<0.01, *p<*0.001) place aversion in the morphine-treated groups versus the saline-treated mice. For the morphine treated groups, the aversion for the naloxone-paired chamber was lower in male (*p*<0.05) and female (*p*<0.01) of CRF1R KO mice versus wild-type mice (Fig 3A). Altogether, these data suggest that male and female of CRF1R KO mice develop less place aversion than wild-type mice.

We examined extinction of CPA score in withdrawn mice for aversion from day 6 to 13. The aversion score are shown in Fig 3B. ANOVA with repeated measures showed significant main effect of sex (F_(1,14)_ = 5.248, *p<*0.03), genotype (F_(1,14)_ = 21.528, *p<*0.0001), time (F_(1,14)_ = 385.544, *p* = 0.0005) and a significant interaction between sex x genotype (F_(1,14)_ = 4.667, *p* = 0.040), time x sex (F_(1,14)_ = 10.291, *p<*0.004) and time x genotype (F_(1,14)_ = 9.693, *p<*0.004). This indicates that the change in aversion score depended on the combined influence of sex x genotype. Wild-type male mice group extinguished their aversion much later than female wild-type. On day 9 both sex of wild-type and male of CRF1R KO mice retained aversion whereas female of CRF1R KO mice significantly (*p<*0.05, *p<*0.01) extinguished its aversion (Fig 3B).

In addition, male CRF1R KO mice extinguished its aversion before wild-type mice (p<0.01). These results indicate that CRF1R KO mice not only showed a lower aversion but also lasted much shorter aversion than wild-type.

### Withdrawal signs

**Fig 4:**
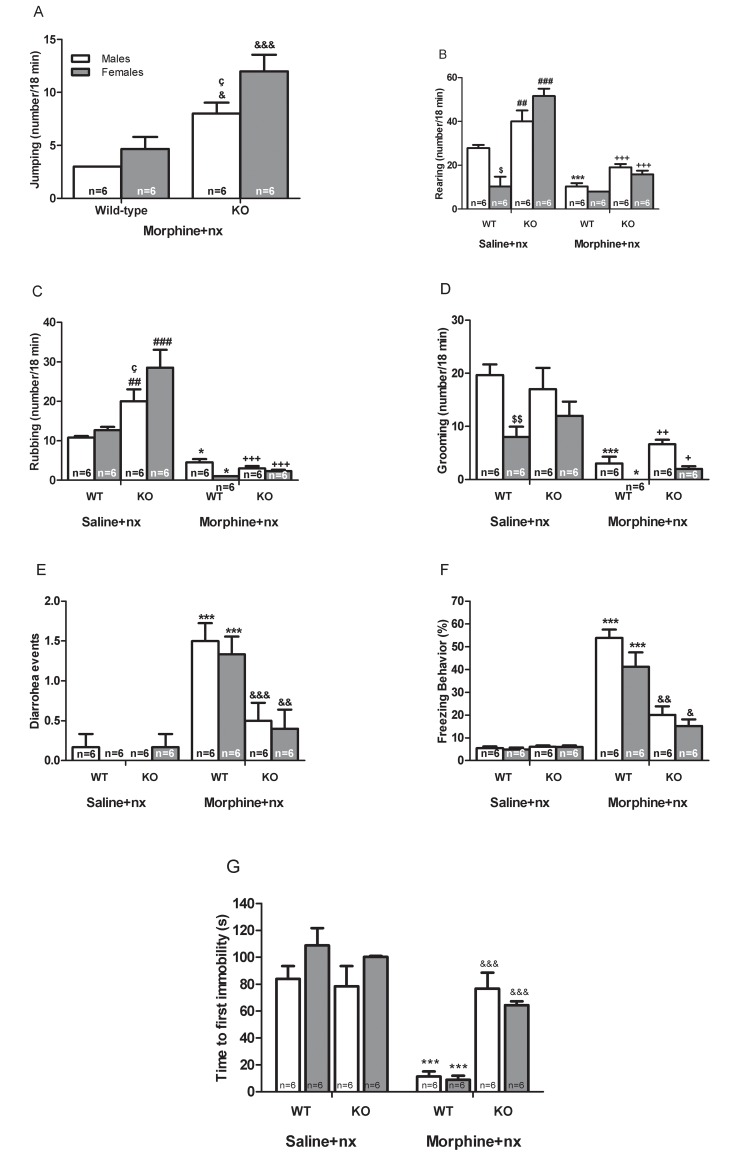
Behaviour effects by naloxone (nx) precipitated morphine withdrawal. We have evaluated the somatic signs: A) jumping, B) rearing; C) rubbing, D) grooming, E) Diarrhoea, and F) Freezing behavior induced after naloxone (1 mg/kg, s.c.)-injection to morphine or saline- treated mice during 18 min. We have also evaluated the time to first immobilization (G). Data are expressed as the mean ± SEM. *p<0.05,***p<0.001 versus WT mice treated with saline+nx; +*p<*0.05, ++*p<*0.01, +++*p<*0.001 versus KO mice treated with saline+nx; &*p<*0.05, &&*p<*0.01, &&&*p<*0.001 versus WT mice treated with morphine+nx; ##*p<*0.01, ###*p<*0.001 versus WT mice treated with saline+nx; $*p<*0.05, $$*p<*0.01 versus male of WT mice treated with saline+nx; çp<0.05 versus female of KO mice treated with morphine+naloxone.

Analysis of somatic opioid withdrawal revealed significant effects of sex [jumping: (F_(1,24)_ = 5.283, *p* = 0.027), and grooming: (F_(1,24)_ = 16.302, *p<*0.0001)], genotype [jumping: (F_(1,24)_ = 21.133, *p<*0.0001), rearing: (F_(1,24)_ = 25.484, *p<*0.0001), rubbing: (F_(1,24)_ = 16.597, *p<*0.0001), and diarrhoea: (F_(1,23)_ = 14.814, *p<*0.0001)], morphine treatment [jumping: (F_(1,24)_ = 125.941, *p<*0.0001), rearing: (F_(1,24)_ = 141.789, *p<*0.0001), rubbing: (F_(1,24)_ = 99.113, *p<*0.0001), grooming: (F_(1,24)_ = 56.465, *p<*0.0001), and diarrhoea F_(1,23)_ = 45.817, *p<*0.0001)], significant interactions for sex x morphine treatment [jumping: (F_(1,24)_ = 5.283, *p* = 0.027), rearing: (F_(1,24)_ = 8.353, *p* = 0.006), and rubbing: (F_(1,24)_ = 4.543, *p* = 0.039)], and genotype x morphine treatment [jumping: (F_(1,24)_ = 21.133, *p<*0.0001), and rubbing: (F_(1,24)_ = 18.402, *p<*0.0001)]. Three-way ANOVA on total percent of time freezing revealed a significant effect of sex (F_(1,24)_ = 4.243, *p* = 0.046), genotype (F_(1,24)_ = 43.173, *p<*0.0001), morphine treatment (F_(1,24)_ = 149.797, *p<*0.0001), significant interactions for sex x morphine treatment (F_(1,24)_ = 5.283, *p<*0.0001), and genotype x morphine treatment (F_(1,24)_ = 48.625, *p<*0.0001). The analysis of time to first immobility showed significant effects of genotype (F_(1,24)_ = 12.288, *p* = 0.001), morphine treatment (F_(1,24)_ = 47.554, *p<*0.0001), sex x morphine treatment (F_(1,24)_ = 4.116, *p* = 0.049), and genotype x morphine treatment interactions (F_(1,24)_ = 19.573, *p<*0.0001). *Post hoc* test showed that CRF CRF1R KO mice made more jumps (*p<*0.05, *p<*0.001) than wild-type mice (Fig 4A). Wild-type and CRF1R KO mice treated with morphine showed decreased rearing (*p<*0.001), rubbing (p<0.05, p<0.001) and grooming (*p*<0.05, *p*<0.01, *p*<0.001) versus the groups treated with saline (Fig 4B, 4C and 4D) However, there was an increase in diarrhoea (*p<*0.001) and freezing (*p<*0.001) in wild-type mice treated with morphine when compared with wild-type mice treated with saline (Fig 4E and 4F). Morphine-withdrawn CRF1R KO male and female had less freezing (*p<*0.05, *p<*0.01) than wild-type mice of the same sex (Fig 4F).

Overall, CRF1R KO mice treated with saline instead morphine showed an increased rearing (*p<*0.01, *p<*0.001), and rubbing (*p<*0.01, *p<*0.001) versus wild-type mice with the same treatment (Fig 4B and 4C). Male and female of CRF1R KO mice treated with morphine presented an increase time to first immobility (*p<*0.001) versus wild-type mice with the same treatment. These results clearly indicate an increase in jumps and in time to first immobility together with decrease in freezing in CRF1R KO mice treated with morphine when compared with wild-type mice.

### ACTH and corticosterone plasma levels

Morphine withdrawal stimulates HPA axis [20] via the release of CRF in the PVN. The activation of CRF1R by CRF induced a common response of elevated ACTH and corticosterone. To evaluate whether a causal link exists between CRF1R activation and HPA axis, we measured plasma ACTH and corticosterone levels in male and female from wild-type and CRF1R KO mice after naloxone-induced CPA expression and CPA extinction. Three way ANOVA for ACTH plasma levels revealed a main effect of sex [extinction: (F_(1,20)_ = 51.590, *p<*0.0001)], genotype [expression: (F_(1,20)_ = 92.441, *p<*0.0001)], treatment [expression: (F_(1,20)_ = 111.101, *p<*0.0001)], sex x genotype [expression (F_(1,20)_ = 5.398, *p* = 0.026)], and genotype x treatment interaction [expression: (F_(1,20_) = 105.355, *p<*0.0001)].

**Fig 5:**
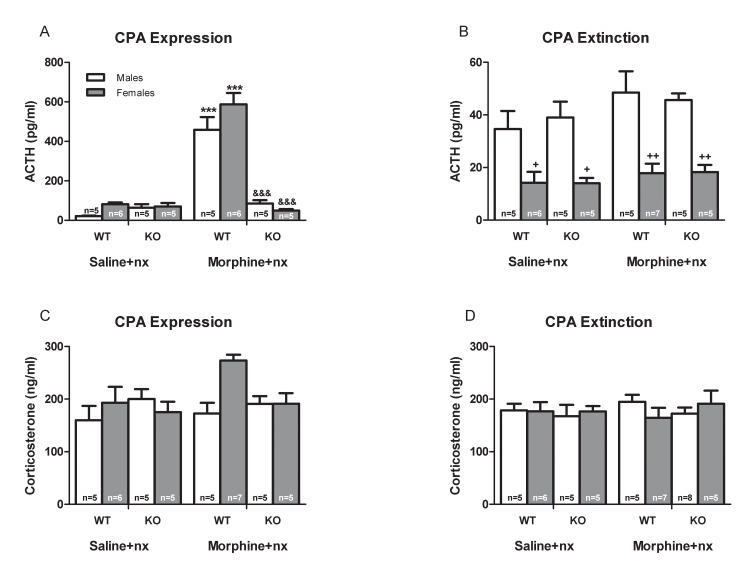
ACTH and corticosterone plasma levels after the expression and extinction of CPA training. ACTH (A,B) and corticosterone (C,D) plasma levels in wild-type (WT) and knockout (CRF1R KO) mice after CPA expression and extinction training. Data are expressed as the mean ± SEM. ***p<0.001 versus WT mice treated with saline+naloxone (nx); &&&p<0.001 versus WT mice treated with morphine+nx; +*p<*0.05, ++*p<*0.01 versus male of WT and CRF1R KO mice.

As shown in Fig 5A and 5B naloxone induced a dramatic increase (*p<*0.001) on ACTH plasma levels in morphine withdrawn male and females wild-type mice after CPA expression. Morphine-treated CRF1R KO males and females injected with naloxone showed a significant (*p<*0.001) decrease in plasma ACTH levels versus wild-type mice. However, no changes in ACTH plasma levels were observed after CPA extinction when compared wild-type and CRF1R KO mice treated with morphine versus the groups treated with saline instead of morphine. Extinct morphine-withdrawn females showed decreased (*p<*0.05, *p<*0.01) in ACTH plasma levels versus males from both genotypes and both treatments (morphine or saline). With respect to corticosterone, three way ANOVA revealed no significant effects of sex, genotype, treatment, sex x genotype, sex x treatment, genotype x treatment or sex x genotype x treatment interaction in CPA expression or CPA extinction (Fig 5C and 5D).

## Discussion

In the present study we demonstrated no significant alteration in some morphine withdrawal signs such as body weight loss, jumping, rearing, rubbing, grooming, diarrhoea, freezing and time to first immobility between male and female wild-type mice. These results establish that equivalent degrees of physical dependence were generated in both sexes. In agreement with the present results, it has been reported that the naloxone-precipitated morphine withdrawal syndrome appeared to be equivalent in both sexes when the antagonist was administered rapidly following the last morphine injection [7]. In addition, when higher doses of morphine (8–120 mg/kg) were administered by injection or osmotic minipump for 3–9 days, no sex differences were observed in naloxone-precipitated morphine withdrawal examined 30 min or 3h after morphine administration in mice [12,37]. In contrast, naloxone-precipitated morphine withdrawal was greater in male than in female rats assessed approximately 1–3 day after the last morphine injection [38]. These discrepancies could be explained by the differences in the morphine regimen dose and differences between species and the mice strains used. Another study in mice demonstrated that some symptoms of spontaneous withdrawal from morphine occurred earlier in males than in females, and body weight loss was greater and longer in males than in females treated with high doses of morphine [39]. There are important differences in behavioural responses and in the molecular adaptive changes between naloxone-precipitated morphine withdrawal and spontaneous morphine withdrawal so it is difficult to compare the present with prior study. In agreement with previous studies [31,40,41] body weight loss and freezing were significantly attenuated in CRF1R KO mice. According to a previous study [19] our results also showed an increase in jumping in CRF1R KO mice similarly to that described previously [19]. Although among withdrawal behaviours in mice, jumping is widely considered the most sensitive and reliable index of withdrawal intensity and is the most commonly used[12,42–45], it should be noted that distinct neural substrates mediate various withdrawal symptoms [46,47]. Thus, findings using naloxone-precipitated jumping in CRF1R KO mice do not easily extrapolate to other parameters of dependence such as body weight loss.

It is commonly accepted that affective drug withdrawal symptoms are of major motivational significance in contributing to relapse and continued drug use; thus, it is important to understand the mechanisms that mediate affective behaviours during morphine withdrawal. A previous work has suggested that nicotine and morphine withdrawal associated with negative affective states and place aversion to previous neutral environmental stimuli, could represent a motivational component in the maintenance of drug of abuse [48]. In the present study, we further investigated the mechanism underlying sex-specific differences in CPA expression and extinction. There are no significant sex differences in wild-type or CRF1R KO mice for the CPA expression. However, CRF1R KO mice presented less aversion than wild-type mice suggesting that CRF1R is implicated in the conditioned place aversion induced by naloxone in morphine-treated mice. However, the body weight loss was significantly higher in female of CRF1R KO mice than males one indicating a stronger withdrawal syndrome in female versus male of CRF1R KO mice.

There is much information about the neurobiological mechanisms of extinction or reward memory of drug taking [49–51]. However, little information is known about extinction of aversive memory of drug withdrawal [52]. We found sex differences between male and female mice for CPA extinction. Thus, CPA expression in male mice was showed until the day 11. This could be mediated by an increased memory consolidation process in male mice during the post-test, although it is difficult to distinguish experimentally between these possibilities. Our results showed that male mice spent more days to finish the CPA extinction compared to females. Probably, gender-related variety in sex steroids and the hormonal effects in the brain may account for this difference in the CPA extinction. In agreement with previous results that demonstrated that naloxone-precipitated withdrawal exerts its motivational effects, as measured by CPA, in a genotype-dependent manner [53] here we clearly demonstrated that the genetic disruption of the CRF/CRF1R pathway decreases the period of CPA extinction in male and female suggesting a role for CRF1R in aversive memory. Memory impairment during drug withdrawal is a complex phenomenon that requires an understanding of the mechanisms underlying extinction of aversive memories, which could lead to pharmacological approaches for enhancing extinction, which might facilitate the treatment of drug addiction. In this study, we have evaluated the role of HPA axis in the CPA expression and extinction in both males and females. Our findings demonstrated that plasma ACTH levels were increased in male and female of wild-type mice, while plasma corticosterone was not changed after CPA expression. Although the presence of pituitary ACTH is clearly essential for adrenocortical function, ACTH-independent mechanisms seem to have an important role modulating the highly sensitive adrenal stress system to adapt its response appropriately to physiological needs. Numerous studies have been published indicating that a large number of neuropeptides, neurotransmitters, growth factors and even bacterial ligands are capable to modulate adrenal glucocorticoids release independently of pituitary ACTH [54]. Adrenocortical cells express a great variety of receptors for these factors, thus enabling potential direct actions on corticoids release. Lesions of upstream stress regulatory pathways in the brain lead to a dissociation between ACTH and corticosterone, suggesting that central nervous system pathways are able to regulate HPA axis function at both the pituitary and adrenal level [55]. Our results also showed that ACTH release observed after CPA expression was attenuated in CRF1R KO mice. According to these results, plasma ACTH levels were found to be decreased in morphine withdrawn animals treated with CRF1R antagonists [40]. In addition, a role for the HPA axis and brain extra-hypothalamic CRF/CRF1R circuitry in somatic, molecular, and endocrine alterations induced by opioid withdrawal has been reported [19]. ACTH plasma levels returned to basal levels in male and female wild-type and CRF1R KO mice after CPA extinction. These results suggest that the CPA expression is at least, partially, due to an increase in plasma ACTH levels which can be decreased after naloxone CPA extinction. Wild-type and CRF1R KO female mice present lower ACTH levels than male after CPA extinction. These data could explain the differences between sexes observed in the extinction of aversive memory.

We have documented differences between males and females in the CPA extinction training. The observed sex differences may be based in part on intrinsic sex differences in the ability to associate (learning) and recall (memory) aversive events. The extent cognitive differences in the ability to store and remember aversive events remain to be elucidated. In this regard, this study demonstrates that CPA expression training increases plasma ACTH levels, which is critical for maintenance of aversive memory associated with drug withdrawal. Our results provide the first evidence that CPA extinction training induced a higher decrease in ACTH plasma levels in female than in male which could be responsible of the differences between sexes observed. This study also demonstrated that CRF1R is involved in the in the expression and extinction of aversive memory induced by naloxone-precipitated withdrawal. Our findings may facilitate the development of treatments for opioid addiction.
